# Ferulic Acid against Cyclophosphamide-Induced Heart Toxicity in Mice by Inhibiting NF-*κ*B Pathway

**DOI:** 10.1155/2016/1261270

**Published:** 2016-01-04

**Authors:** Yafan Song, Chunyan Zhang, Congxia Wang, Ling Zhao, Zheng Wang, Zhijun Dai, Shuai Lin, Huafeng Kang, Xiaobin Ma

**Affiliations:** ^1^Department of Cardiology, The Second Affiliated Hospital, Medical School of Xi'an Jiaotong University, Xi'an 710004, China; ^2^Department of Oncology, The Central Hospital of Xi'an, Xi'an 710003, China; ^3^Department of Oncology, The Second Affiliated Hospital, Medical School of Xi'an Jiaotong University, Xi'an 710004, China

## Abstract

The purpose of the present study was to elucidate the protective effects of ferulic acid (FA) against cyclophosphamide- (CTX-) induced changes in mice. Forty-eight male ICR mice were divided into four groups. Control group was intraperitoneally (i.p.) injected with 200 *μ*L of phosphate buffer saline (PBS). Model group was intraperitoneally injected with a single dose of CTX (200 mg/kg). FA (50 mg/kg) and FA (100 mg/kg) groups were intraperitoneally injected with a single dose of CTX (200 mg/kg) followed by the intragastric treatment with FA (50, 100 mg/kg) for 7 consecutive days. After 12 d, the mice were sacrificed to analyze the hematological, biochemical, histological parameters and mechanism research. The results indicated that FA significantly decreased the serum levels of alanine aminotransferase (ALT), aspartate aminotransferase (AST), creatine kinase (CK), lactate dehydrogenase (LDH), interleukin-6 (IL-6), IL-1*β*, and tumor necrosis factor-*α* (TNF-*α*) in CTX-injected mice. In addition, FA effectively reduced the total numbers of white blood cells (WBCs), red blood cells, platelets, and hemoglobin content. FA also obviously attenuated the histological changes of the heart tissues caused by CTX. Moreover, Western blot demonstrated that FA inhibited the phosphorylations of NF-*κ*B signaling pathway in CTX-stimulated cardiac tissues. In conclusion, FA might be considered as an effective agent in the amelioration of the heart toxicity resulting from CTX treatment.

## 1. Introduction

Cyclophosphamide (CTX) is a frequently used anticancer chemotherapeutic agent used alone or in a combination with other medicines for the treatment of several human malignancies [1]. Because of its immunosuppressive activity, CTX could also be used in preconditioning the host for immunotherapy [2]. Evidence has emerged indicating that the administration with CTX increased the numbers of the dendritic cells which directly drove the immune response and also activated the immune reaction by promoting the mobilization of the hematopoietic stem cells [3, 4]. Despite many beneficial effects, several adverse side effects of CTX have been reported, such as pneumonitis, pulmonary fibrosis [5], bone marrow suppression [6], the induction of genotoxicity [7], and heart toxicity [8]. In particular, the incidence of the cardiotoxic effects of CTX treatment still remains high. Exposure to high dose CTX leads to the acute cardiotoxic effects including myocyte damage, extravasation of toxic metabolites, and diastolic contractile dysfunction [9, 10]. Herein, the injection of CTX could act as an experimental model in our study.

NF-*κ*B is a key transcriptional factor involved in inflammatory progression of various diseases [11]. Accumulating evidence indicated the pivotal role of NF-*κ*B in cardiac disorder [12]. It is well known that the IKK complex mediates the phosphorylation and degradation of I*κ*B, which consequently promotes NF-*κ*B molecule [13]. The activation of NF-*κ*B contributes to the productions of inflammatory cytokines and the modulations of other biochemical indices of heart disease [14].

Ferulic acid (4-hydroxy-3-methoxycinnamic acid), a cinnamic acid derivative, is one of the large families of biologically active substances in fruits, vegetables [15], and medicinal herbs [16]. Many former investigations have shown that ferulic acid acts as a free radical scavenger including hydroxyl and peroxyl radicals [17] or an inhibitor of lipid peroxidation [18]. As to our knowledge, there are few available reports associated with the protective effect of FA on CTX-induced alterations. Therefore, the present study was aimed at addressing the ameliorated effect and exploring the protective mechanism of FA on CTX-challenged heart toxicity.

## 2. Materials and Methods

### 2.1. Chemicals

CTX and FA were purchased from Sigma-Aldrich (St. Louis, MO). ALT, AST, and CK kits were produced by Nanjing Jiancheng Bioengineering Institute (Nanjing, China). LDH, IL-6, IL-1*β*, and TNF-*α* ELISA commercial kits were supplied by BioLegend (San Diego, CA, USA). All antibodies were provided by Cell Signaling Technology.

### 2.2. Animals and Treatment

48 ICR mice (8-week-old, 18–22 g) were purchased from Jiangning Qinglongshan Animal Cultivation Farm (Nanjing, China). The animals were maintained under standard laboratory conditions (temperature 24 ± 2°C and natural light-dark cycle) and had free access to drinking water and standard pellet diet. After acclimatization for one week to laboratory conditions, the mice were randomly divided into four groups. Control group was intraperitoneally (i.p.) injected with 200 mL of phosphate buffer saline (PBS). Model group was intraperitoneally injected with a single dose of CTX (200 mg/kg). FA (50 mg/kg) and FA (100 mg/kg) groups were intraperitoneally injected with a single dose of CTX (200 mg/kg) followed by the intragastric treatment with FA (50, 100 mg/kg) for 7 consecutive days. After 12 days, the mice were sacrificed to analyze the hematological, biochemical, histological parameters and mechanism research.

### 2.3. Hematological Profile

Cardiac function parameters included red blood cells (RBCs) counts, white blood cells (WBCs) counts, the total platelets, and hemoglobin content (Hb g/dL), and counts were measured from fresh blood samples obtained from the orbital plexus of the eyes of all groups at the end of the experiment using the electronic blood counter. Differential WBCs were carried out from blood smears on days 0, 2, 4, 8, and 12.

### 2.4. Biochemical Assays

At the end of the experiment, the blood was harvested from each sacrificed mouse and centrifuged at 3 000 r/min for 10 min. Serum levels of ALT, AST, CK, and LDH were assayed according to the instructions of commercial kits (Nanjing Jiancheng Bioengineering Institute, Nanjing, China).

### 2.5. Cytokine Measurement in Serum

The concentrations of IL-6, IL-1*β*, and TNF-*α* in serum were detected with ELISA kit according to the manufacturer's instructions.

### 2.6. Histopathological Analysis of Heart

Immediately after the sacrifice of the anesthetized mice, the heart tissues were quickly removed and fixed in 10% formalin solution for more than 48 h. For the histological examinations, the samples were dehydrated in graded alcohol, embedded in paraffin wax, and stained with hematoxylin-eosin (H&E). After that, pathological changes were examined by light microscopy for observation of structural abnormality.

### 2.7. Western Blot Analysis

Proteins in heart tissues were extracted with lysis buffer (RIPA with protease and phosphatase inhibitor) for 30 min on ice and then centrifugated at 12000 rpm for 5 min at 4°C. The concentration of total protein was determined by enhanced bicinchoninic acid (BCA) protein assay commercial kit (Beyotime, Nanjing, China). Equal amounts of protein were subjected to the 8–12% SDS-polyacrylamide gel electrophoresis and transferred to polyvinylidene fluoride membranes (Millipore Corporation, MA, USA). The blots were incubated with the appropriate concentration of specific antibodies overnight at 4°C. Then, the membranes were blocked in skim milk and treated with horseradish peroxidase-conjugated second antibody for 1 h at room temperature. Immunoreactivity was detected with an ECL Key-GEN system by a gel imaging system (ChemiScope 2850, Clinx Science Instruments Co. Ltd., Shanghai, China). The quantification of protein expression was determined using a densitometer (Imaging System).

### 2.8. Statistical Analysis

All data in the figures are expressed as means ± SDs and analyzed with GraphPad by one-way analysis of variance (ANOVA) with Tukey multiple comparison test. *P* < 0.05 was considered statistically significant.

## 3. Result

### 3.1. Effects of FA on Serum Biochemical Parameters

**Figure 1:**
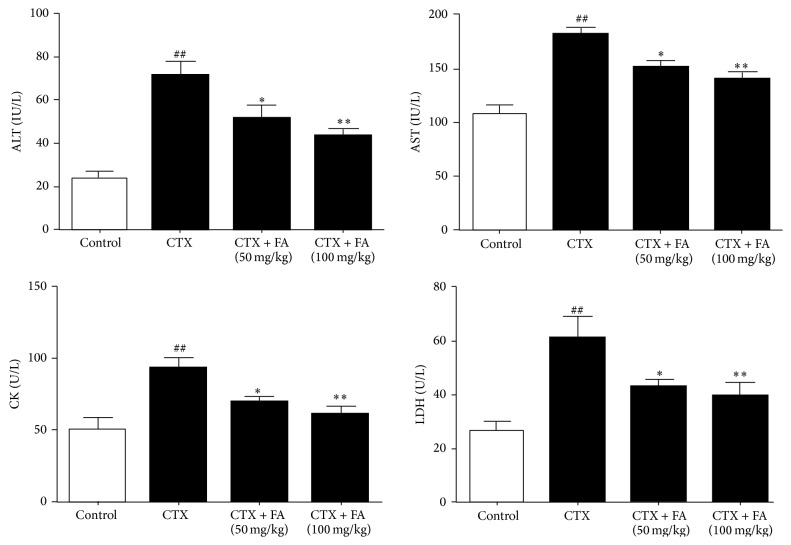
Effects of FA on serum biochemical parameters. All values given are the mean ± SD. *P* < 0.05 and ^##^ *P* < 0.01 versus control group. ^*∗*^ *P* < 0.05 and ^*∗∗*^ *P* < 0.01 versus CTX group.

Figure 1 summarizes the levels of critical biochemical parameters in acute heart toxicity, such as ALT, AST, CK, and LDH. Relative to those in control group, CTX induction caused dramatic increases in the activities of ALT, AST, CK, and LDH. On the contrary, the treatment with FA in CTX administered rats remarkably suppressed all the activities of these enzymes, clearly suggesting that FA is capable of inhibiting the levels of biochemical parameters in CTX-stimulated heart toxicity.

### 3.2. Effects of FA on Hematological Parameters

#### 3.2.1. Total Number of WBCs

**Figure 2:**
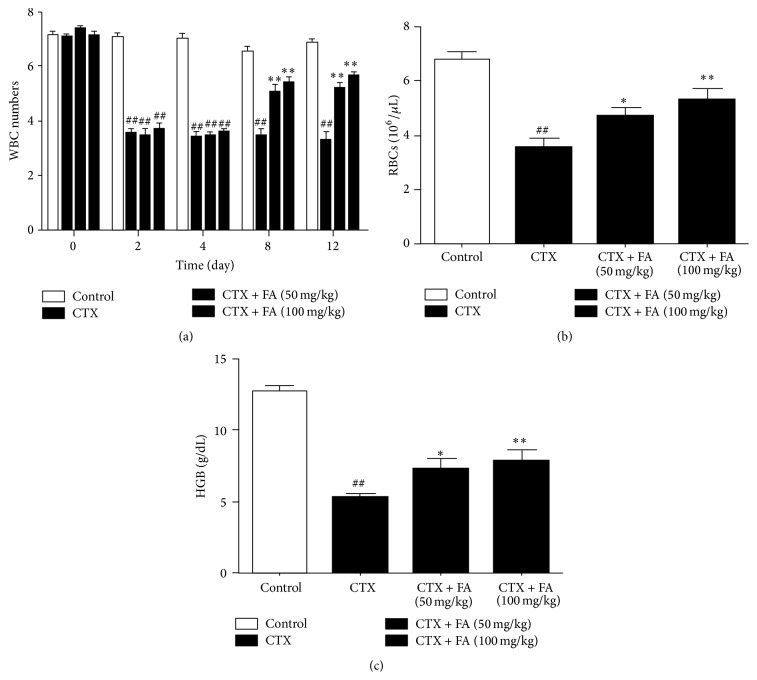
Effects of FA on hematological parameters. All values given are the mean ± SD. *P* < 0.05 and ^##^ *P* < 0.01 versus control group. ^*∗*^ *P* < 0.05 and ^*∗∗*^ *P* < 0.01 versus CTX group.

WBC is the critical part of immune system and plays the essential role in the host defense. From the study (Figure 2(a)), the total number of WBCs was significantly decreased in CTX-injected mice compared with that in the control group from day 8 to day 12; the mice which were injected with CTX along with FA showed a remarkable increase in the WBCs counting compared with that in CTX injected mice from day 8 to day 12.

#### 3.2.2. Total Number of Red Blood Cells (RBCs) and Hemoglobin Level

To evaluate the oxygen carrying capacity, we detected the number of RBCs and the level of hemoglobin. As shown in Figure 2(b), CTX stimulation causes the dramatic decreases in the number of red blood cells (RBCs) and hemoglobin level in CTX-injected mice compared with those in control group. However, treatment with FA effectively increased RBC counts and compared hemoglobin content compared with those in the CTX-injected mice.

#### 3.2.3. Total Number of Blood Platelets

It is widely acknowledged that CTX conduces to the decrease of blood platelets. Thus, we counted the number of platelets to confirm the protective effects of FA on CTX-induced cardiotoxicology. As revealed in Figure 2(c), the total number of blood platelets was obviously decreased in CTX-injected mice compared with that in control group, while administration with FA significantly increased blood platelets compared with that in the mice exposed to CTX.

### 3.3. Effects of FA on Serum Inflammatory Cytokines

**Figure 3:**
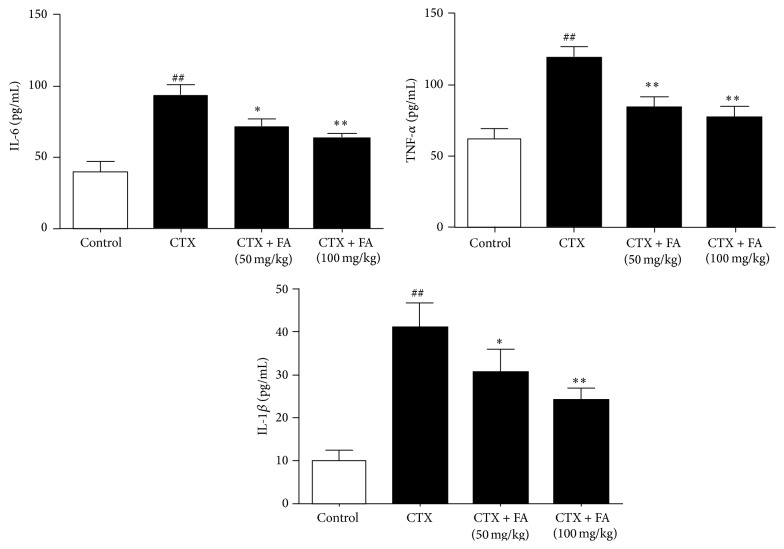
Effects of FA on serum inflammatory cytokines. All values given are the mean ± SD. *P* < 0.05 and ^##^ *P* < 0.01 versus control group. ^*∗*^ *P* < 0.05 and ^*∗∗*^ *P* < 0.01 versus CTX group.

The inflammatory cytokines including TNF-*α*, IL-1*β*, and IL-6 have been recognized as the indicator of the inflammatory response during the heart toxicity.  Figure 3 displayed a marked increase in the levels of IL-6 and TNF-*α* in the CTX-injected mice compared with those in control mice. On the contrary, treatment with FA (50, 100 mg/kg) showed the significant decreases in these values compared with those in CTX-injected mice. These data suggested that FA participated in the synthesis and release of inflammatory cytokines of CTX-induced heart toxicity.

### 3.4. Effect of FA on the Histopathological Picture of the Heart Tissues

**Figure 4:**
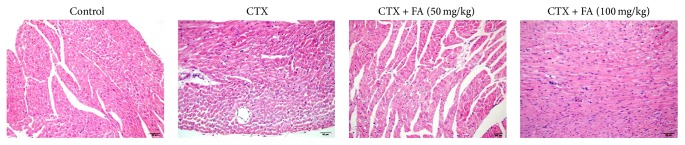
Effect of FA on the histopathological picture of the heart tissues. All values given are the mean ± SD. *P* < 0.05 and *P* < 0.01 versus control group. *P* < 0.05 and *P* < 0.01 versus CTX group.

Histopathological observations revealed no appreciable alterations in the control group. On the other hand, heart sections from mice that were stimulated with CTX showed that heart degeneration and infiltration of lymphocytes were observed (Figure 4), while examination of the heart tissues from the mice revealed that treatment with FA obviously ameliorated heart degeneration and infiltration of lymphocytes. The results clearly indicated that FA attenuated the histopathology condition of CTX-stimulated heart toxicity.

### 3.5. Effects of FA on NF-*κ*B Pathway in Heart Tissues

**Figure 5:**
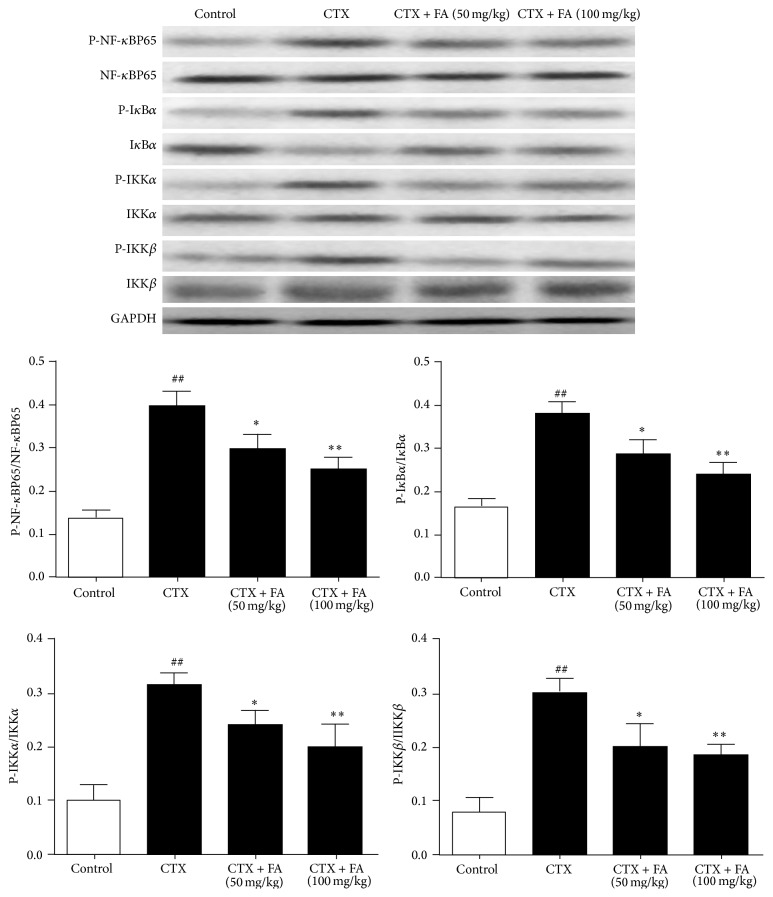
Effects of FA on NF-*κ*B pathway in heart tissues. All values given are the mean ± SD. *P* < 0.05 and ^##^ *P* < 0.01 versus control group. ^*∗*^ *P* < 0.05 and ^*∗∗*^ *P* < 0.01 versus CTX group.

To characterize the cardioprotective-related signaling pathway of FA-treated mice, the phosphorylated and nonphosphorylated forms of the NF-*κ*B pathway components were detected. Western blot analysis showed that the exposure to CTX remarkably upregulated the levels of p-NF-*κ*Bp65, p-I*κ*B*α*, p-IKK*α*, and p-IKK*β* compared with those in the control group. Notably, the administration with FA blocked the phosphorylations of NF-*κ*Bp65, I*κ*B*α*, IKK*α*, and IKK*β* in the CTX-injected mice. Our analytical results demonstrated that the inhibitory effects of phosphorylated IKK complex caused by FA might contribute to the degradation and of I*κ*B*α* leading to the activation of NF-*κ*Bp65 in heart tissues with LPS challenge (Figure 5).

## 4. Discussion

The present study showed that FA exhibited protective effects on CTX-induced heart toxicity in mice. FA significantly inhibited the serum levels of ALT, AST, CK, LDH, IL-6, IL-1*β*, and TNF-*α*. In addition, FA effectively decreased the hemoglobin level and total numbers of WBCs, RBCs, and blood platelets and attenuated the histological change. Western blot analysis demonstrated that FA ameliorated CTX-challenged heart toxicity possibly through the IKK/I*κ*B/NF-*κ*B signaling pathway.

Previous work investigated the therapeutic effects of FA at the doses of 50 and 100 mg/kg; herein, we detected the pharmacological activity of FA (50, 100 mg/kg) in the present study [19]. FA exerted anti-inflammatory, hepatoprotective, and antivirus properties [20]. Several pieces of evidence displayed that FA exhibited cardioprotective effects on coronary heart disease [21] and myocardial ischemia-reperfusion injury [22]. In addition, it was noteworthy that FA showed the beneficial effects on isoproterenol-induced myocardial infarction [23] and *β*-adrenergic catecholamine induced cardiotoxicity [24]. However, whether FA exhibits a potent therapeutic activity against CTX-stimulated heart toxicity remains poorly defined.

As a commonly used alkylating agent, the cyclophosphamide (CTX) has been usually used for the intervention of neoplastic disease, such as leukemia, lymphomas, and brain cancer. It is also reported that CTX increases the proportion of myeloid derived suppressor cells in blood and lymphoid organs and suppresses the function of the immune system to fight against cancerous cells [25]. However, heart toxicity is one of the major side effects of CTX and contributes to a high rate of morbidity and mortality [26]. Frequent literatures focused on CTX-induced acute fulminant congestive heart failure and hemorrhagic myopericarditis [27]. Previous investigator studied the cardiac injury with the injection of CTX [28]. In the present work, we employed this model to evaluate the protective effect of FA on CTX-induced heart toxicity.

The numbers of blood platelets, WBCs, and RBCs and the level of hemoglobin are the sensitive blood tests applied to the diagnosis of the cardiac function in heart disease. In CTX-challenged group, the enhanced hematological parameters were clearly evidenced by marked increases in the above indices. By contrast, FA provoked protection against CTX through the inhibitions of blood platelets, WBCs, and RBCs numbers and hemoglobin content. Together with the obvious attenuation of histopathologic condition in heart tissue, these findings confirmed that FA effectively ameliorated the cardiotoxic effects caused by CTX injection.

LDH is the specificity enzyme in the cytoplasm and releases into blood during cardiotoxic dysfunction. Additionally, CK distributes in the myocardium and is widely considered as the contributing factors for heart disease [29]. AST and ALT are the critical transaminases which mediate the cardiac metabolism. It was reported that the injection with CTX remarkably exaggerated the toxicity in hearts of mice evidenced by the upregulations of LDH, CK, AST, and ALT [30]. The present study confirmed these phenomenons and further displayed that FA could ameliorate the lesion of heart toxicity through the inhibitions of these indices.

Thus, we investigated the possibility that treatment with FA inhibited the production of IL-1*β*, IL-6, and TNF-*α* via the promotion of NF-*κ*B. Generally, IL-1*β* participates in the repair of acute inflammatory disorder [31]. IL-6 is responsible for expanding the inflammatory cascade in the pathogenesis of inflammatory process [32]. TNF-*α* plays an important role in stimulating the secretion of other inflammatory mediators and motivating innate immune response [33, 34]. As expected, treatment with FA (50, 100 mg/kg) significantly suppressed the overproductions of inflammatory cytokines caused by CTX-challenged cardiotoxicity.

To further verify the nature of the inhibitory effect of FA on the generation of inflammatory cytokine, we detected the effects of FA on the recruitment of the IKK/I*κ*B-*α*/NF-*κ*B signaling pathways. It is widely acknowledged that NF-*κ*B signaling pathway is essential to the generations of inflammatory cytokines induced by CTX. IKK complex consists of IKK-*α* and IKK-*β*, which are both the primary regulators involved in the NF-*κ*B pathway [35]. The suppression of phosphorylated IKK*α*/*β* consequently conduced to the phosphorylation and degradation of I*κ*B-*α*, followed by the activation of NF-*κ*B [36]. NF-*κ*B is considered to be a major target for the treatment of cardiotoxic response [37]. According to our knowledge, the promotion of NF-*κ*B results in the upregulation of proinflammatory cytokines including TNF-*α*, IL-6, and IL-1*β* [38]. Accumulating evidence indicated the essential role of NF-*κ*B in cardiac dysfunction. Kis et al. elicited that NF-*κ*B played a crucial role in the ischaemia reperfusion [39]. Upregulation of NF-*κ*B activity has been also proved to mediate oxidative stress and inflammatory condition in cardiac hypertrophy mice [40]. It was also demonstrated that the activation of NF-*κ*B was highly related to the pathogenesis of cardiac fibrosis [41]. Notably, previous literature proposed that cyclophosphamide-induced cardiac injury was closely associated with the inflammatory cytokines generation which was widely considered to be governed by NF-*κ*B [30]. Our analytical results suggested that FA could suppress the phosphorylations of IKK/I*κ*B/NF-*κ*B signaling.

In conclusion, the present study demonstrated that the FA administration improved cardiac function after CTX-stimulated heart toxicity in mice. The cardioprotective effect of FA might attribute to its ability of suppressing biochemical indicators and inhibiting inflammatory cytokines, which possibly partially occurred via the inhibition of the IKK/I*κ*B/NF-*κ*B pathway. Therefore, our experimental results suggested that FA could be potential therapeutic medicine for the treatment of cardiovascular disorder. Further studies are warranted to explore the clinical application of FA in the future.
